# P-1237. Vancomycin Population Pharmacokinetics and Toxicity-Exposure Relationships in Children with Multiple Organ Dysfunction Syndrome

**DOI:** 10.1093/ofid/ofaf695.1429

**Published:** 2026-01-11

**Authors:** Justin Shiau, Victor Amajor, Sylwia Marianski, Nathaniel J Rhodes, Amanda Bwint, Anna Sharova, Mark Hall, Manjunath P Pai, Bo Wen, Kevin J Downes, Marc H Scheetz

**Affiliations:** Midwestern University Pharmacometrics Center of Excellence, Downers Grove, IL; Children's Hospital of Philadelphia, Philadelphia, Pennsylvania; Midwestern University, Downers Grove, IL; Midwestern University, Downers Grove, IL; Children's Hospital of Philadelphia, Philadelphia, Pennsylvania; Children's Hospital of Philadelphia, Philadelphia, Pennsylvania; Nationwide Children's Hospital, Columbus, Ohio; University of Michigan, Ann Arbor, MI; University of Michigan, Ann Arbor, MI; Children's Hospital of Philadelphia, Philadelphia, Pennsylvania; Midwestern University, Downers Grove, IL

## Abstract

**Background:**

Vancomycin (VAN) is a first line antibiotic for severe gram-positive bacterial infections in pediatrics but is associated with exposure-dependent kidney injury. In children with multiple organ dysfunction syndrome (MODS), most initial VAN dosing is guided via weight-based approaches. We developed a population pharmacokinetic (popPK) model for children with MODS to evaluate the relationship between acute kidney injury (AKI) and VAN exposures (area under the concentration-time curve [AUC]).

**Table 1.**
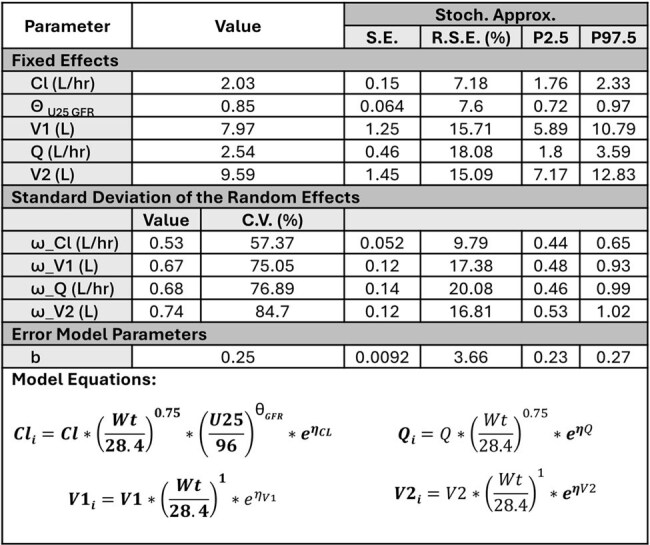
Population PK parameter fixed effects estimates and random effects (i.e. between subject variability) for central volume of distribution(V1), clearance (CL), beta coefficients on weight adjusted (individual weight/28.4kg) for V1 and CL, peripheral volume of distribution (V2), and intercompartmental bi-directional flow (Q).

**Figure 1.**
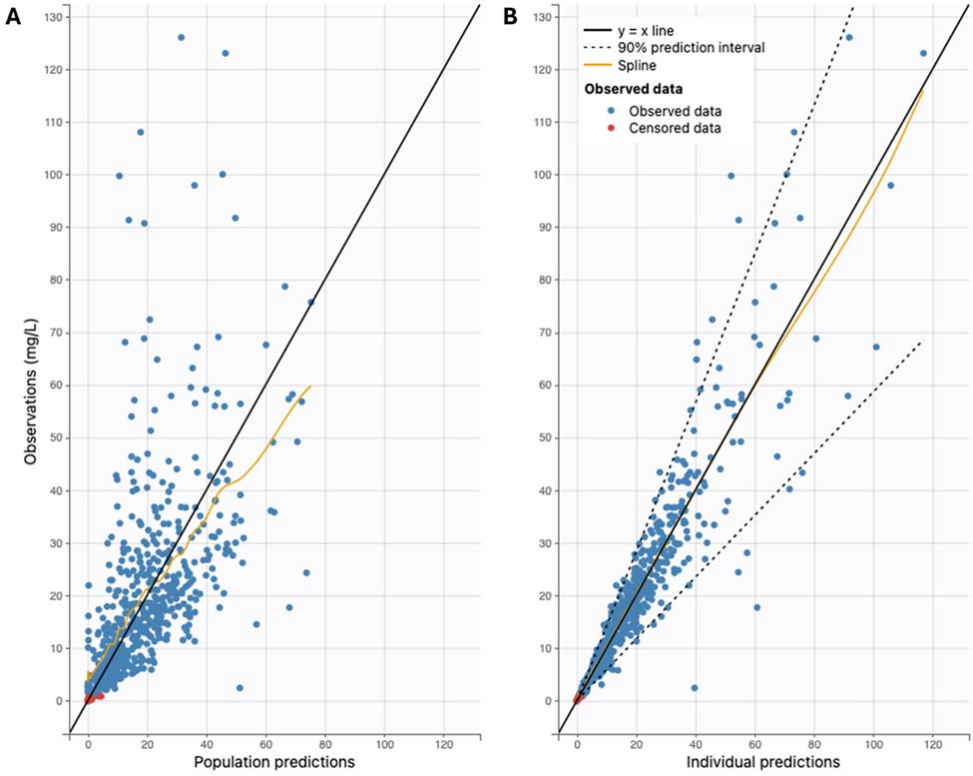
Observed vs. (A) population and (B) individual predictions for the model.

**Methods:**

We conducted a multi-center prospective observational PK study (AMPLE) embedded in a larger study of critically ill children with MODS (PARADIGM). Up to 15 PK samples were collected via volumetric absorptive microsampling over 3 days. Parametric popPK modeling was performed using Monolix 2024R1. Covariate inclusion was based on objective function and physiologic relevance. VAN AUC_0-24_ and AUC_24-48_ for each child were calculated using Empiric Bayes Estimates in Simulx 2024R1. AKI was considered a 0.3 mg/dL or 50% increase from ICU baseline. Sensitivity and specificity for classifying AKI at each AUC was calculated. A stepwise multivariate analysis determined factors (including AUC) associated with AKI.

**Figure 2.**
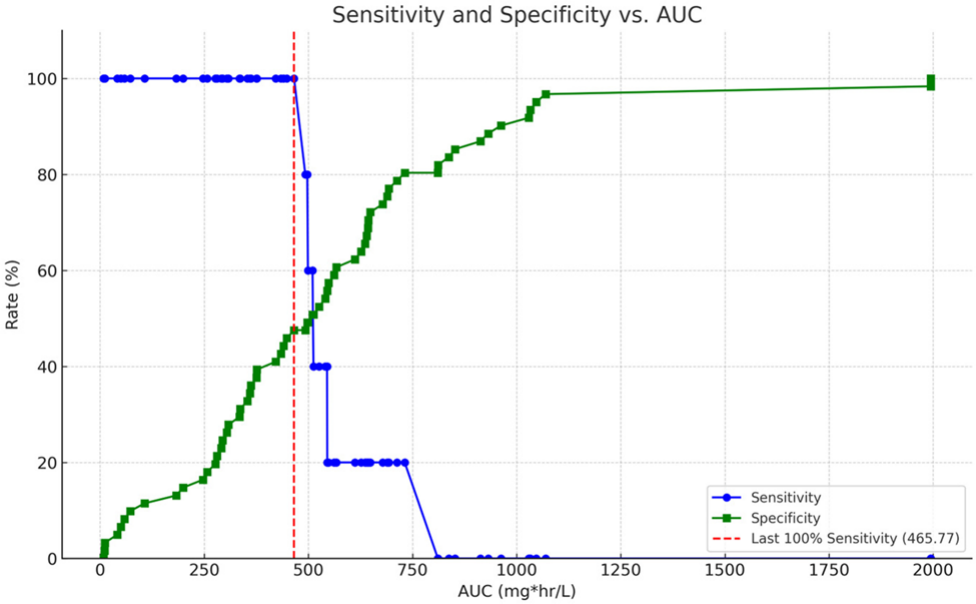
Sensitivity and specificity rates of specific AUC values.

**Table 2.**
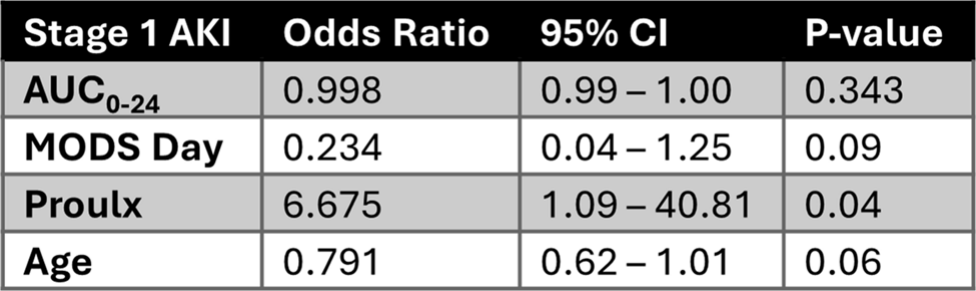
Logistic regression analysis for stage 1 AKI.

**Results:**

66 subjects were included (median age of 10 y [range: 1 m – 17 y], median weight of 30 kg [range: 3 – 214]). A two-compartment model with clearance adjusted for allometric scaling (weight^0.75^) and glomerular function (calculated using CKiD Under 25 [U25] equation) best described the data (Table 1, Fig. 1). Median AUC_0-24_ and AUC_24-48_ were 454 mg*hr/L (range: 194-1569) and 505 (range: 8-1994), respectively. 7 total subjects met criteria for ICU-emergent AKI. An AUC_24-48_ of 465.8 mg*hr/L maintained the best sensitivity (100%) whereas at AUCs ≥ 545.5 mg*hr/L sensitivity dropped to ≤ 20% (Fig. 2). In the multivariate analysis only the Proulx score for classifying MODS was significant for AKI (6.68 OR, 95% CI 1.1 – 40.8, Table 2).

**Conclusion:**

Children with MODS had widely varying AUCs. AUCs ≥ 465.8 best identified AKI. In children with MODS, ICU-emergent AKI occurred within the current targeted therapeutic range. AUCs should be maintained as low as feasible.

**Disclosures:**

Nathaniel J. Rhodes, PharmD MS, Apothecademy, LLC: Advisor/Consultant Mark Hall, MD FCCM, Abbvie: Advisor/Consultant|Kiadis: Licensing income unrelated to this submission|Partner Therapeutics: Partner Therapeutics provides study drug for a clinical trial for which I am PI. This trial is unrelated to the submitted abstract|Sobi: Sobi provides study drug for a clinical trial for which I am PI. This trial is unrelated to the submitted abstract Kevin J. Downes, MD, Paratek Pharmaceuticals, Inc.: Grant/Research Support|Veloxis Pharmaceuticals, Inc.: Grant/Research Support Marc H. Scheetz, PharmD, MSc, Doseme: Advisor/Consultant|other: Additional not relevant to this abstract. If more information is needed about unrelated relationships, I can provide it.

